# Inanition

**Published:** 1852-01

**Authors:** 


### INANITION.

Dr. London, of West Point, Tenn., in a letter some time since received, mentions an instance of prolonged inanition in a hog, which deserves a place by the side of the one recorded by Martell. The unfortunate animal, according to Dr. L., became confined between the limbs of a tree—wedged in—and in this situation remained for ninety-six days in the autumn and winter of 1848—'9. During this period it had no food, but, it may be supposed, was pretty abundantly supplied with rain water. Its computed weight at the time that it became entangled was 190 lbs., and but 30 lbs. when released, so that it lost 160 lbs. in 96 days. The pig mentioned by Martell as having been inhumed by a slip from the cliffs of Dover, lived one hundred and sixty days without food, and was found to have diminished in weight in that time more than 120 lbs. The more rapid emaciation in the former case was doubtless induced by the exposure of the animal to the open air and the vicissitudes of the weather. The pig under the chalk cliff was effectually protected from these influences, haviug just a sufficiency of air to maintain respiration. Dr. Currie relates an interesting case of dysphagia, in which the subject being unable to take any food for a month, lost during that time 100 lbs. in weight. The patient complained very little of hunger; neither was he much disturbed by thirst except during the first days of his abstinence, and found that it was then always removed by a tepid bath Nutritious clysters were employed and seemed to support his strength as well as afford him refreshment. It is remarkable how little this patient suffered at any period of his inanition. "His nights," says Dr. Currie, "were quiet; his sleep sound and apparently refreshing." Towards the end of it he had very lively dreams, but all of a pleasant nature. "No man," remarks Currie, "ever perhaps approached death by steps more easy."

A child in this city died inanitiated about a year since, from closure of the esophagus, the result of swallowing a solution of pearlash. The obstruction was gradual, and the child lingered for more than three months. We had an opportunity in this case of remarking the fetid odor of which writers speak as emitted by the bodies of those in the last days of starvation. With this little sufferer, as with the patient of Dr. Currie, life ebbed so gradually away, that its mother was hardly aware of the moment when it ceased to breathe.

